# Assessment of Cardiovascular Fibrosis Using Novel Fluorescent Probes

**DOI:** 10.1371/journal.pone.0019097

**Published:** 2011-04-20

**Authors:** Jiqiu Chen, Seung Koo Lee, Wael R. Abd-Elgaliel, Lifan Liang, Elisa-Yaniz Galende, Roger J. Hajjar, Ching-Hsuan Tung

**Affiliations:** 1 Cardiovascular Research Center, Mount Sinai School of Medicine, New York, New York, United States of America; 2 Department of Radiology, The Methodist Hospital Research Institute, Weill Medical College of Cornell University, Houston, Texas, United States of America; Heart Center Munich, Germany

## Abstract

Cardiovascular fibrosis resulted from pressure overload or ischemia could alter myocardial stiffness and lead to ventricular dysfunction. Fluorescently labeled collagen-binding protein CNA 35, derived from the surface component of *Staphylococcus aureus*, and a novel synthetic biphenylalanine containing peptide are applied to stain fibrosis associated collagen and myocytes, respectively. Detailed pathological characteristics of cardiovascular fibrosis could be identified clearly in 2 hours. This staining pair requires only simple staining and brief washing, generating less than 10 ml of waste. The image information collected by this novel fluorescent staining pair is compatible with it collected by the traditional Masson's Trichrome and Picrosirius Red staining which are widely used to stain cardiovascular fibrosis and isolated cells.

## Introduction

Fibrosis in the heart is associated with many cardiac diseases, such as hypertrophy and coronary ischemic heart disease.[1], [2] To assess the dynamic status of fibrosis, which is an alternation of myocardial collagen network, proper reagents that recognize collagen fibers *in vitro* and *in vivo* would be extremely valuable in understanding the definitive mechanism of hypertrophy and heart failure. Currently histological analysis of heart tissues is the most accepted and sensitive way to study pathological changes of fibrosis. Masson's Trichrome and Picrosirius Red are two commonly used collagen staining methods for cardiovascular fibrosis detection.[3], [4] They provide clear contrast to distinguish collegens from surrounding connective tissue and cells. Although these methods are excellent in examining collagen density and structure, the procedure is lengthy and consumes large amount of reagents and washing solutions. Cleaner and more efficient histopathological stains, which could offer compatible histological details while conserving reagents, resource and time, would certainly be a favorable choice.

**Figure 1:**
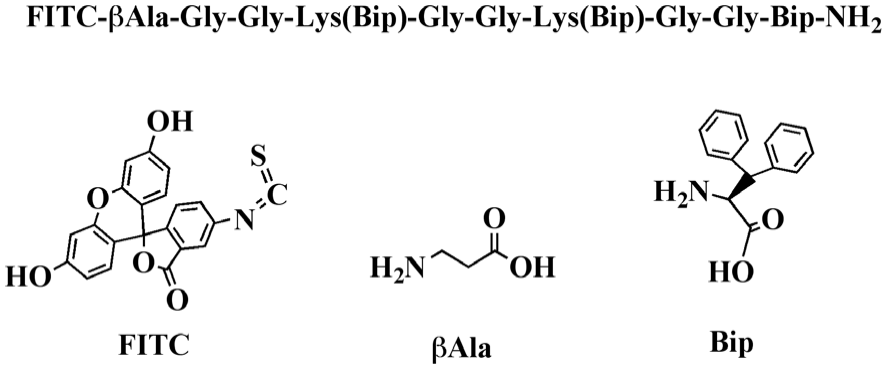
Chemical structure of MTP.

Recently we have found two new probes which are suitable for pathological analysis of fibrotic tissues. The first probe, termed CNA35, is derived from the collagen binding microbial surface components recognizing adhesive matrix molecules (MSCRAMM) on *Staphylococcus aureus.* [5] The original CNA protein is composed of two subdomains (N_1_ and N_2_), each adopting an IgG-like fold. According to recently reported crystal structure of N_1_N_2_ segment of CNA, it forms a complex with synthetic collagen-like triple helical peptide.[6] CNA35, a truncated form of CNA with a higher affinity than the wild type protein, has been used as a tissue staining probe based on its specific binding property to collagen fibers, including collagen type I, III and IV.[7] In animal studies, CNA35 has recently been applied to label collagen of atherosclerotic arteries in healthy and apolipoprotein E^−/−^ mice.[8], [9], [10] The second probe, termed myocyte targeting peptide (MTP) probe, is a short synthetic peptide which contains three biphenylalanine (Bip) unusual amino acid residues (Figure 1). Its excellent selectivity for myocyte was discovered serendipitously during the development of collagen binding probes.

In present study, we demonstrated the usage of CNA35 and MTP probes in assessing myocardial fibrosis induced by aortic constriction following coronary arterial occlusion and aorta de-banding. Comparing with Masson's Trichrome and Picrosirius Red, this “green” staining pair presents significant advantages in staining excised tissues and isolated cells, while offering compatible staining with little amount of agents and in a short period of time.

## Results

### Tissue preparation and general staining procedures

After 5 minutes fixation with 50% methanol + 50% acetone, the CNA35/MTP staining could be conveniently done on OCT samples with a 60-min incubation at room temperature and followed by three simple washes, all by pipetting small amount of reagents and buffers. The overall procedure for probe staining took about 2 hours, and the total volume of waste is less than 10 ml; while the conventional Masson's Trichrome and Picrosirius Red stain on OCT sections took about the same time, but required few more steps of staining and washing.

### Comparing with conventional Picrosirius Red and Masson's Trichrome staining in histological sections

**Figure 2:**
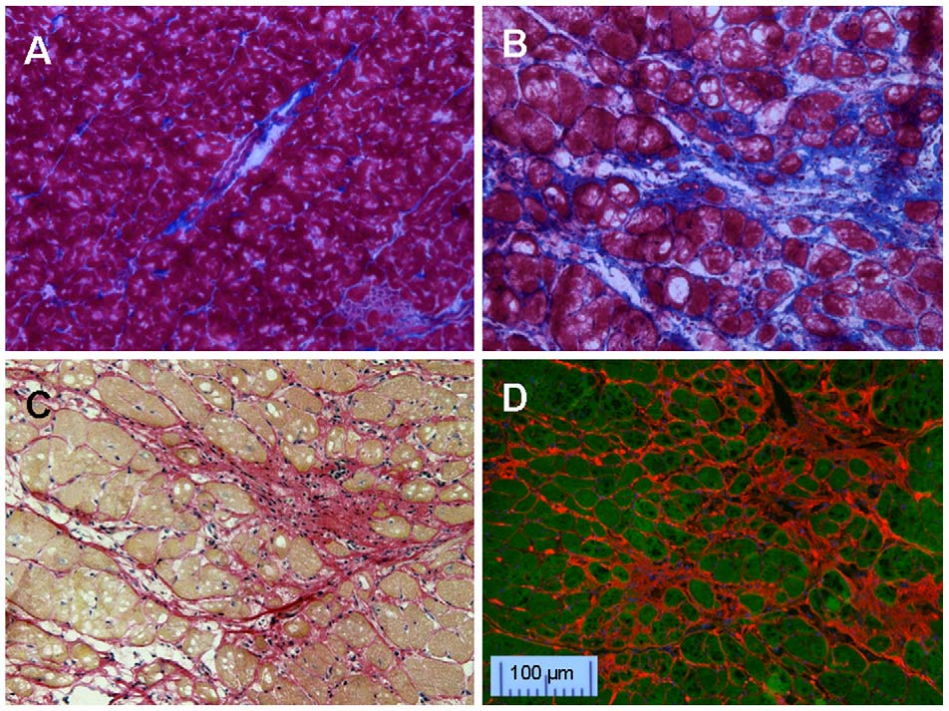
Interstitial fibrosis in hypertrophic hearts. A. normal control heart and B. hypertrophic heart with Masson's Trichrome stain. Blue  =  fibrous collagen, red  =  myocytes, black  =  nuclei, C: Picrosirius Red stain on hypertrophic heart, red  =  fibrous collagen, pale yellow  =  myocytes, black  =  nuclei. D: CNA35-Cy3 (red) and MTP-FITC (green) double stain on hypertrophic heart.

After Masson's Trichrome staining, distinct blue collagen fibers and red myocytes were observed in the tissues collected from hypertrophic hearts. (Figure 2A and B). Similarly, Picrosirius Red staining (Figure 2C) was able to demonstrate the fibrosis and nucleus in myocytes nicely. CNA35 fluorescent staining also highlighted interstitial fibrosis with similar quality (Figure 2D). With a counterstain of myocyte with FITC labeled MTP, the structure of collagens was clear.

**Figure 3:**
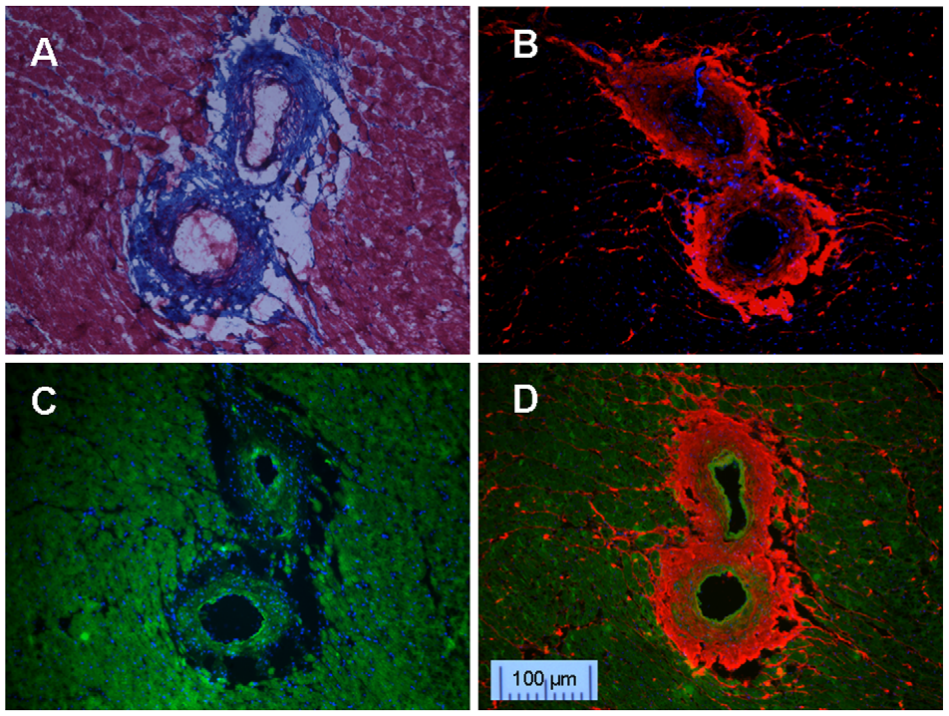
Perivascular fibrosis staining. A: Masson's Trichrome staining. B: CNA35 mono staining for collagen fibers. C: MTP mono staining for myocyte. D: Double staining for collagen (CNA35-Cy3, red) and myocyte (MTP-FITC, green).

The networked collagens are usually strong in vessel stress, but their local tissue density is relatively low. After fixing by Bouin's solution (containing 10% formalin) during Masson's Trichrome staining, the perivascular tissue became shrunken, resulting in obvious defects (empty spots) in the perivascular space (Figure 3A). When tissue sections were fixed with 50% methanol + 50% acetone, CNA35 monochrome staining showed more intact collagen fibers compared with Masson's Trichrome staining (Figure 3B). Although the vessel borders were difficult to define by CNA35 staining alone, MTP was able to clearly stain myocytes and thickened arterial wall, offering a useful counterstain (Figure 3C). Therefore, dual staining with CNA35 and MTP provided excellent delineation of perivascular fibrosis and vascular wall (Figure 3D).

**Figure 4:**
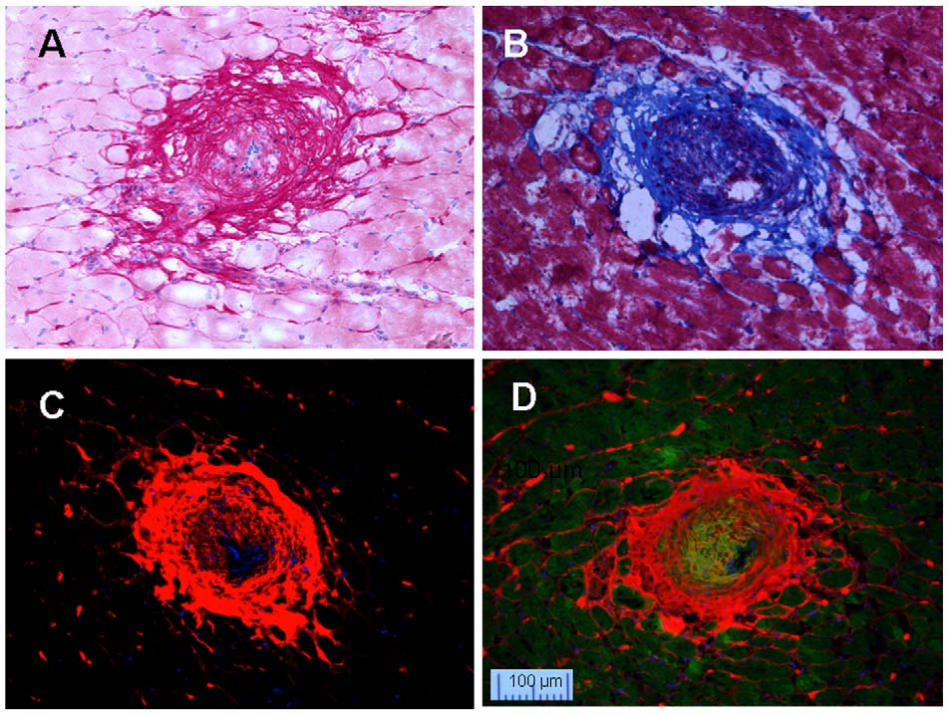
Staining of neointima in coronary arteries in Ab+I/R+DeAb heart in rats. A: Picrosirius Red staining. B: Masson's Trichrome. C: CNA35 mono staining. D: Double staining for collagen (CNA35-Cy3, red) and myocyte (MTP-FITC, green).

**Figure 5:**
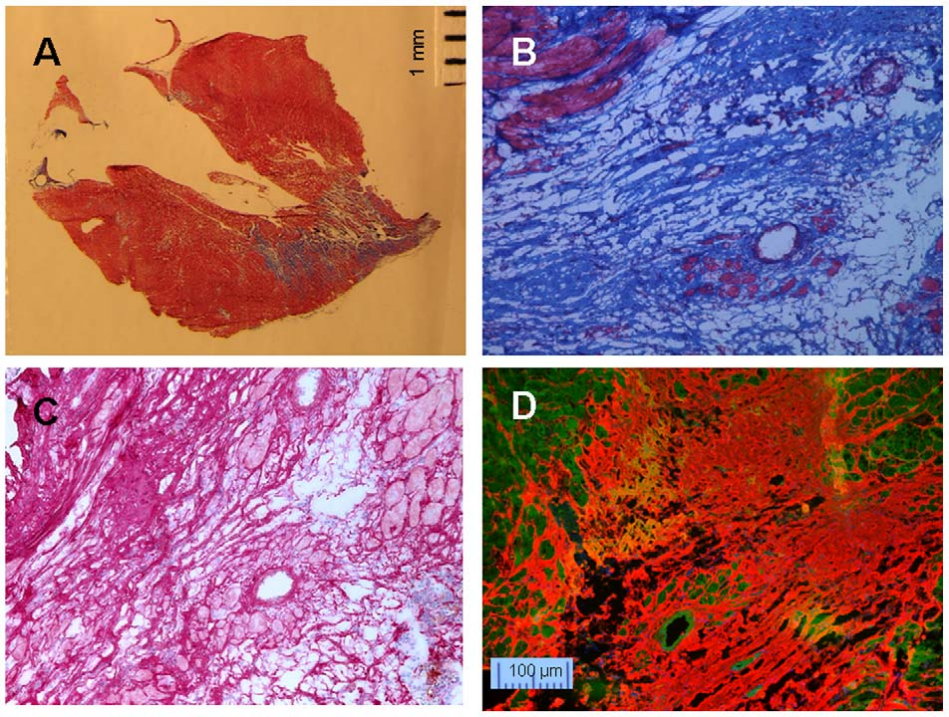
Staining of fibrosis in infarcted area. A and B: Masson's Trichrome, C: Picrosirius Red staining. D: Double staining for collagen (CNA35-Cy3, red) and myocyte (MTP-FITC, green).

Neointima resulted from aortic banding plus ischemia/reperfusion followed by aortic de-banding (Ab+I/R+DeAb) was shown by Picrosirius Red staining (Figure 4A) and Masson's Trichrome (Figure 4B) stain, respectively. In comparison, greater detail of the neointimal components could be seen using CNA35/MTP double labeling. Figure 4C shows MTP mono-staining and Figure 4D shows fibrillar matrix under collagens by dual staining. Similar observation was obtained with myocardial infarct tissues (Figure 5). Masson's Trichrome staining was good for general slide staining (Figure 5A and 5B), and Picrosirius Red was excellent in showing cellular nuclei (Figure 5C). Complex collagen network in the area after myocardial infarction was probed clearly by CNA35 (Figure 5D). These dual florescent images suggested that the CNA35/MTP double labeling kit could offer greater detail of tissue architecture in a region of fibrosis.

### Fluorescent imaging on isolated myocytes and fibers

**Figure 6:**
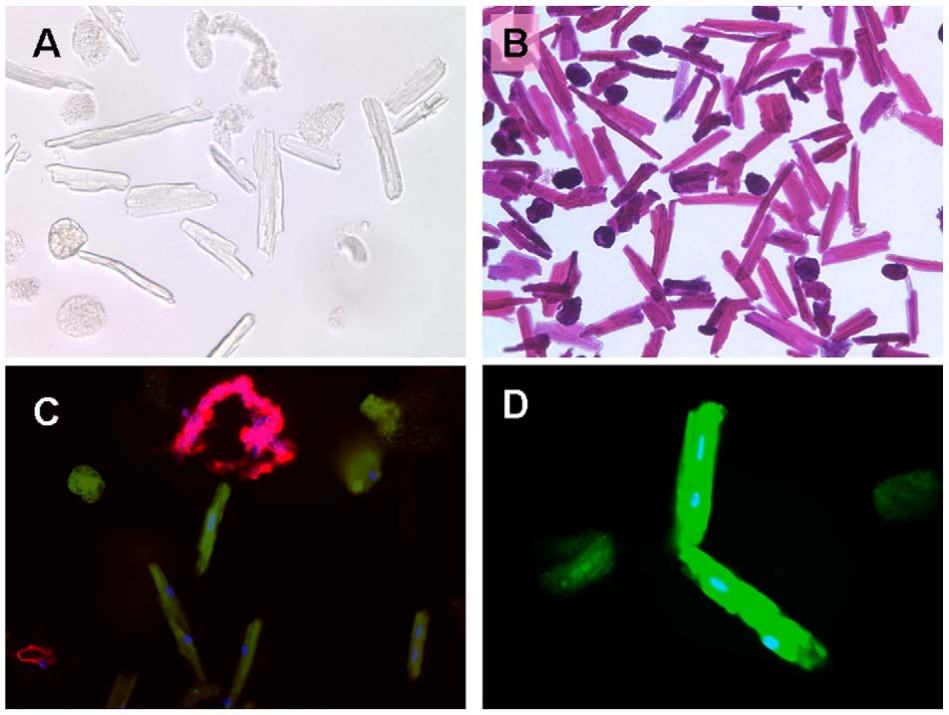
Images of isolated myocytes and fibers. A: Bright-field image of myocytes without staining. B: Bright-field image of myocytes with H-E staining. C: Double staining for collagen (CNA35-Cy3, red) and myocyte (MTP-FITC, green). Florescent probe could distinguish fibers from alive and dying (round) myocytes. D: Enlarged myocytes labeled with MTP-FITC. Blue is DAPI for nuclei.

To further explore their potential applications, CNA35 and MTP fluorescent probes were tested on freshly isolated myocytes and fibers. Without staining, it is difficult to define collagen fibers from dead cells under bright-field microscopy (Figure 6A). Hematoxylin and eosin staining was likewise limited (Figure 6B). However, CNA35 and MTP double labeling could clearly differentiate live fibers (Figure 6C, red), dying cells (Figure 6C, green, round) and live myocytes (Figure 6C and 6D, green, rod cells).

## Discussion

The myocardium is an elastic network of myocytes enmeshed in a collagen matrix that connects the myocytes and supporting coronary vasculature. The adaptive remodeling process consists of a range of changes in tissue architecture, which include cardiovascular fibrosis, vessel wall thickening, endothelial dysfunction and myocyte hypertrophy.[11] The response of individual myocardial components is influenced by both hemodynamic and nonhemodynamic factors.[12], [13], [14] Fibrosis is initially a reparative response to high blood pressure or myocardial infarction.[11] It is important to have an effective and rapid technique to assess dynamic fibrosis in the infarcted scar and remote myocardium.

CNA35, a small protein extracted from the collagen-binding domain of *Staphylococcus aureus*, preserves the high collagen binding specificity and affinity.[5] Recently CNA35 with a fluorescent tag has been applied to visualize collagen not only in tissue and live cell culture, but also in an animal atherosclerosis model.[7], [8], [10], [15] In conjunction with MTP, a new myocyte staining agent, we confirmed that CNA35/MTP is an excellent pair in probing cardiovascular fibrosis on heart tissue slides and isolated cardiac collagen fibers.

CNA35/MTP staining is a simple and fast technique which uses frozen sections, produces very little (10 ml) potential hazardous waste and can be completed in less than 2 hours. This staining protocol is compatible with traditional Masson's Trichrome and Picrosirius Red stainings which are often used to measure cardiovascular fibrosis. If Picrosirius Red or Masson's Trichrome stain was performed on frozen OCT-embedded sections, the whole procedure also takes about 2 hours. Whereas, if paraffin sections were used, the staining procedures, including deparaffinization, rehydration and dehydration, produce more than 1 liter of highly hazardous, carcinogenic chemicals (including formaldehyde, picric acid, and hematoxylin) and more than 10 liters of diluted toxic aqueous waste due to extensive washing, and take more than 6 hours to complete.

Pathophysiology of isolated myocytes has been studied for decades,[16], [17] while isolated cardiac collagen fibers has yet to be investigated. Upon isolation, collagen fibers and dead myocytes could not be distinguished easily *in vitro* under a regular microscope. Our staining presents a promising way to study the elastic characteristics of viable cardiovascular fiber bundles *in vitro*.

We have also demonstrated the superb myocyte selectivity of the MTP probe in both tissues section and live cells. It is not clear how MTP probe could selectively bind to myocyte with such high affinity, but this selectivity appears to be Bip dependent (data not shown). Our preliminary experiments showed that MTP peptides with fewer Bip had much less affinity to myocytes. More studies are in progress to understand this distinct binding selectivity.

In this study, FITC and Cy3 were used as the fluorescent reporters. Many more commercially available fluorochromes with different optical properties could be selected according to the needs. Furthermore, these two probes could be used conveniently in conjunction with other fluorescently labeled antibodies to assess the interactions between collagen fibers, myocytes, vessel endothelium and other pathologically relevant structures or molecules. Although histological staining could provide details tissue information, it still relies on ex vivo analysis on collected tissues. If an imaging technology could provide similar information non-invasively, it will be extremely useful in clinical diagnosis. Currently we are in the process of labeling CNA35 and MTP with imaging reporters, and then will try to image cardiovascular fibrosis using animal models.

In conclusion, CNA35 and MTP are new and promising fluorescent probes in the assessment of cardiovascular fibrosis. CNA35/MTP double-staining is a simple and fast method with minimal environmental toxic waste to enable co-labeling of collagen fibers and myocytes in hypertrophy and coronary ischemic disease *in vitro*.

## Materials and Methods

### Collagen binding CNA35 probe

Recombinant CNA35 protein was a kind gift from Dr. Magnus Hook (Texas A&M University, USA). CNA35 (1 mg) in sodium carbonate-sodium bicarbonate buffer (1 ml) was reacted with Cy3 monofunctional reactive dye (GE Healthcare, Piscataway, NJ, USA) for 30 min at room temperature. Cy3-labelled CNA35 (CNA35-Cy3) were purified by gel permeation chromatography after pre-equilibrating the sephadex G25 (GE Healthcare) column with phosphate-buffered saline. The final concentration and degree of labeling were determined by measuring the absorbance spectrum of the labeled protein by UV-visible spectrophotometer (Varian, Palo Alto, CA, USA). On average, CNA35-Cy3 tagged ratio was 0.8.

### Biphenylalanine Myocyte targeting peptide (MTP) probe

FITC βAla-Gly-Gly-Lys(Bip)-Gly-Gly-Lys(Bip)-Gly-Gly-Bip-NH_2_ was synthesized by solid-phase peptide synthesis on an automatic synthesizer (ABI-433A, Applied Biosystems, Carlsbad, CA, USA). Briefly, the C-terminal Bip (Synthetech, Albany, OR, USA) was directly attached to resin support by standard Fmoc strategy. After elongation of the peptide chain, fluorescein 5-isothiocyanate (FITC) was coupled to the last *N*-terminal amino acid, beta alanine (βAla). For subsequent side chain modification, the MTT protecting group of the lysine residues was selectively removed with 1% TFA in DCM. Thereafter, Bip was coupled to the Lys side chains using HOBt/HBTU/DIPEA (Figure 1). The peptide was cleaved and purified through reversed phase high performance liquid chromatography (RP-HPLC) in a linear gradient from 0% B to 60% B (8 ml/min) in 70 minutes. The purity and identity of the purified MTP was confirmed by LC-MS on C-18 Vydac column, 5 µm, 4.6 mm ID×150 mm L, (GRACE, Deerfield, IL, USA) using Accela HPLC coupled with photodiode array detector and autosampler (ThermoFisher Scientific, West Palm Beach, FL, USA). Fractions with the same purity (≥95%) were collected together and lyophilized to yield yellowish powders. ESI-MS: calculated: C_93_H_100_ N_16_O_17_S = 1745.95; observed: (M+H)^+^  = 1746.70; (M+2H)^2+^/2 = 873.42 and (M+3H)^3+^/3 = 582.66. The conjugate were stored at 4°C in the dark.

### Animal Protocol

All procedures conformed to the recommendations of the Guide for the Care and Use of Laboratory Animals (Department of Health and Human Services publication number NIH 78–23, 1996) and were approved by the Mount Sinai School of Medicine Animal Care and Use Committee. Sprague Dawley male rats (200 g) underwent aortic constriction for four months to induce hypertrophy and interstitial and perivascular fibrosis. The ascending aorta was banded (Ab) with a 4-0 suture against a PE-50 tube (outside diameter  = 0.96 mm) through right thoracotomy at the second intercostal space. In order to induce neointima in coronary artery, two months post aortic banding, the rats underwent LAD ligation for 30 minutes followed by reperfusion for one month.[18] One month after ischemia/reperfusion (I/R) injury, the chest was reopened for aortic de-constriction (DeAb). The suture around ascending aorta was cut with micro-dissection scissors and separated with cotton tipped applicators. One month post de-banding aorta, the hearts were harvested for experiments. For all surgical procedures, anesthesia was induced by intraperitoneal administration of pentobarbital (60 mg/kg).

### Histological Analysis

At end time point, hearts were perfused with 30 ml of cold PBS with 0.1 ml of 1% heparin. LV was harvested, embedded in OCT, frozen and stored at −80°C. OCT heart sample was sectioned at 8 µm. Masson's Trichrome was conducted according to the guideline of the agent kit from Sigma (HT15, St. Louis, MO, USA). Briefly, frozen slides were fixed in Bouin's solution. After incubation in Weigert's Iron Hematoxylin Solution, the slides was stained with Biebrich Scarlet-Acid Fuchsin and Aniline Blue and dehydrated in ethanol and xylene. Extensive washes were done between each staining. The collagen fibers were stained blue, the nuclei were stained black and myocardium was stained red. Picrosirius Red staining was performed following the protocol of the agent kit from Polysciences Inc (cat#: 24901, Warrington, PA, USA). The collagen fibers were stained red, nuclei were blue, and myocytes were pale yellow.

For fluorescent probe staining, frozen sections were fixed with 50% methanol + 50% acetone, blocked with 5% BSA for 30 minutes. The tissues were incubated with one or two fluorescent probes (300 µl/slide, 10 µM solution) for 1 hour at room temperature. After washing three times in PBS, the slides were mounted with DAPI medium (H-1500, Vector Lab, Burlingame, CA, USA). The FITC and Cy3 fluorescent signals were visualized using confocal microscopy.

### Isolation of myocytes and fibers

Rat heart was retrogradely perfused for 5 minutes with oxygenated Krebs-Henseleit buffer at 37°C. The heart was digested by collagenase (type II, Worthington Lakewood, NJ, USA) and hyaluronidase (type II, Sigma).[19] The digestion solution was filtered through a 150-µm steel mesh. Cells were incubated with fluorescent probes (1∶100) and DAPI (1.5 µg/ml) for 30 minutes. The FITC and Cy3 fluorescent signals were visualized using confocal microscopy.
